# Data for teenagers' stressor, mental health, coping style, social support, parenting style and self-efficacy in South China

**DOI:** 10.1016/j.dib.2020.105202

**Published:** 2020-01-25

**Authors:** Xueming Chen, Tour Liu, Jie Luo, Shixiu Ren

**Affiliations:** a Key Research Base of Humanities and Social Sciences of the Ministry of Education, Academy of Psychology and Behavior, Tianjin Normal University, Tianjin, China; b Faculty of Psychology, Tianjin Normal University, Tianjin, China; c Center of Collaborative Innovation for Assessment and Promotion of Mental Health, Tianjin Normal University, Tianjin, China; d School of Educational Science, Guizhou Normal University, Guiyang, China

**Keywords:** Stressor, Mental health, Coping style, Social support, Parenting style, Self-efficacy

## Abstract

Data provided in this article were collected from 3784 high school students in South China, which measured teenagers' stressor (Stressors Scale for Middle School Students, SSMSS), mental health (Symptom Check-List 90, SCL90), coping style (Simplified Coping Style Questionnaire, SCSQ), social support (Social Support Scale, SSS), parenting style (Egna Minnen av Barndoms Uppforstran-own memories of parental rearing practice in childhood, EMBU) and self-efficacy (General Self-Efficacy Scale, GSES). All the instruments for data collection were in the Chinese version. Participants were 3784 students recruited from 15 high schools in Shenzhen, Guangdong Province of South China with random cluster sampling method. Among them, there were 1987 boys and 1797 girls, with an average age of 14.6 and a standard deviation of 1.82. In addition, a.csv file consists of all the variables and questionnaires we used (both in Chinese and in English) are included as a supplementary material. For a discussion of the major finding based on the data please see the article which used a part of questionnaires and participants we supplied in the data set: The relationship between high school students' social support and coping styles: The mediating role of self-efficacy (https://doi.org/10.3969/j.issn.1007-3728.2014.10.016) [1].

**Table:** Specifications Table

Subject area	*Psychology*
More specific subject area	*Psychology (General)*
Type of data	*Microsoft Excel Comma Separated value document(.csv)*
How data was acquired	*Questionnaires*
Data format	*Raw, Analyzed.*
Experimental factors	*The score of each item and the total score of each dimension.*
Experimental features	*The variables were stressor, mental health, coping style, social support, parenting style and self-efficacy.*
Data source location	*Shenzhen, Guangdong Province, South China*
Data accessibility	*Data and questionnaires we used (both in Chinese and in English) are provided as supplementary material.*
Related research article	The relationship between high school students' social support and coping styles: The mediating role of self-efficacy https://doi.org/10.3969/j.issn.1007-3728.2014.10.016 [1]

**Table:** 

**Value of the Data** • These data set provided information on Chinese students' stressor, mental health, coping style, social support, parenting style and self-efficacy, allowing researchers to explore the relationship among them. • These data were collected from high school students in Shenzhen, Guangdong Province, South China. Researchers could learn about the differences of the same variables among different cultural groups. To some extent, it may promote the communication of different cultures. • These data could be used in the construction of Structural Equation Model (SEM), the analysis of Item Response Theory (IRT) and Meta-Analyses to compare and combine the results from different researches.

## 1 Data

The .csv file we supplied presents the data of stressor (including subscales: Learning stress, Teacher stress, Family environment stress, Parenting style stress, Classmates and friends stress, Social and culture stress and Physical and psychological stress), mental health (including subscales: Somatization, Obsessive-compulsive, Interpersonal sensitivity, Depression, Anxiety, Hostility, Phobic anxiety, Paranoid ideation and Psychoticism), coping style (including subscales: Positive coping style and Negative coping style), social support (including subscales: Subjective support, Objective support and Support utilization), parenting style (including two parts: Father's parenting style and Mother's parenting style) and self-efficacy of high school students in Shenzhen, Guangdong Province, South China. Besides, the data was collected during the period of 2011–2014. And we have provided both the Chinese-version questionnaires and the questionnaires in English as supplementary files. Due to the large data set, missing values or incomplete data distributed in different questionnaires, so we uploaded all the data of the subjects without replacing or deleting the missing values. We thought that it was beneficial to do so, because researchers could select variables and process the missing values according to their own research purposes. In addition, if you want to know the basic information of the sample population and the results of descriptive statistics or correlations among all the variables in the data set please see Table 1, Table 2, Table 3, Table 4, Table 5, Table 6. And for a discussion of the major finding based on the data please see the article which used a part of questionnaires and participants we supplied in the data set: The relationship between high school students' social support and coping styles: The mediating role of self-efficacy (https://doi.org/10.3969/j.issn.1007-3728.2014.10.016) [1].

**Table 1:** Frequency (Grade).

	Frequency
7th grade	754
8th grade	1169
10th grade	914
11th grade	947
Total	3784

**Table 2:** Frequency (Lodging or not).

	Frequency
lodging	1238
non – lodging	2546
Total	3784

**Table 3:** Frequency (Only child or not).

	Frequency
only child	1810
not the only child	1974
Total	3784

**Table 4:** Frequency (Family composition).

	Frequency
single-parent family	256
two-parent family	3528
Total	3784

**Table 5:** Descriptive Statistics results.

	*N*	Minimum	Maximum	*Mean*	*SD*
**1.** Stressor	3771	0	133	28.67	18.91
**2.** SCL_90	3773	0	360	57.36	50.56
**3.** Positive coping style	3784	0	36	19.62	6.66
**4.** Negative coping style	3784	0	24	9.57	4.72
**5.** Social support	3783	17	85	64.60	13.86
**6.** Father's positive parenting style	3586	19	76	49.35	12.11
**7.** Mother's positive parenting style	3644	19	76	51.40	11.98
**8.** Father's negative parenting style	1870	42	133	70.99	15.74
**9.** Mother's negative parenting style	1861	42	146	72.85	15.52
**10.** Self-efficacy	3784	1.00	4.00	2.53	0.64

**Table 6:** Correlation matrix of all variables.

	1	2	3	4	5	6	7	8	9	10
**1.** Stressor	1.00	.71**	–.08**	.28**	–.29**	–.22**	–.19**	.40**	.42**	–.12**
**2.** SCL-90		1.00	–.09**	.36**	–.32**	–.20**	–.16**	.36**	.39**	–.13**
**3.** Positive coping style			1.00	.26**	.41**	.34**	.37**	−.02	–.04	.34**
**4.** Negative coping style				1.00	.02	–.01	.01	.20**	.25**	–.03*
**5.** Social support					1.00	.45**	.43**	–.22**	–.26**	.17**
**6.** Father's positive parenting style						1.00	.81**	–.25**	–.27**	.26**
**7.** Mother's positive parenting style							1.00	–.22**	–.27**	.29**
**8.** Father's negative parenting style								1.00	.74**	–.02
**9.** Mother's negative parenting style									1.00	–.03
**10.** Self-efficacy										1.00

*Note*: *p < .05, **p < .01.

## 2 Experimental design, materials, and methods

### 2.1 Participants

The data presented in the article were collected from 3784 high school students in Shenzhen, Guangdong Province, South China. Among them, there were 1987 boys and 1797 girls, with an average age of 14.6 and a standard deviation of 1.82. Participants were recruited with random cluster sampling method. 15 high schools took part in and 3 classes were selected randomly from each grade (3 from 7th grade, 3 from 8th grade or 3 from 10th grade, 3 from 11th grade). In this data set, some variables such as grade, age, gender, lodging or not, only child or not and the family composition were including in it. Specifically, Grade was coded 1 for 7th grade, 2 for 8th grade, 4 for 10th grade and 5 for 11th grade. Gender was coded 0 for male and 1 for female. Lodging or not was coded 0 for lodging and 1 for non-lodging. Only child or not was coded 0 for only child and 1 for not the only child. Family composition was coded 0 for single-parent family and 1 for two-parent family. These variables described the information of the participants in detail and if you want to know the basic information of the sample population please see Table 1, Table 2, Table 3, Table 4.

### 2.2 Questionnaires

#### 2.2.1 Stressors Scale for Middle School Students (SSMSS)

Stressor was measured by the 39-item Stressors Scale for Middle School Students (Chinese version, [2]). This scale reflected the stressful life events that high school students often encounter in their daily life, consisting of seven subscales: Learning stress (5 items), Teacher stress (7 items), Family environment stress (5 items), Parenting style stress (4 items), Classmates and friends stress (7 items), Social and culture stress (6 items), Physical and psychological stress (5 items). Items were measured on a 5-point Likert scale (0 = no effect, 1 = slight effect, 2 = moderate effect, 3 = severe effect, 4 = extremely severe effect). The reliability and validity of this scale were good. The results of Exploratory Factor Analysis showed that the subscales were unidimensional. And in this data set, Cronbach's α of the scale was 0.924.

#### 2.2.2 Symptom Check-List 90 (SCL90)

Mental health was measured by the 90-item Symptom Check-List 90 (Chinese version, [3]), This scale involved nine dimensions: Somatization (12 items), Obsessive-compulsive (10 items), Interpersonal sensitivity (9 items), Depression (13 items), Anxiety (10 items), Hostility (6 items), Phobic anxiety (7 items), Paranoid ideation (6 items), Psychoticism (10 items) and 7 additional items that were not part of the nine dimensions. Items from this scale were rated on a 5-point Likert scale (0 = never, 1 = slight, 2 = moderate, 3 = heavy, 4 = serious). Through this scale, individuals could conduct self-evaluation of their mental health. Besides, SCL-90 could also be used as a method for doctors to evaluate patients' mental health. In this data set, Cronbach's α of the scale was 0.977.

#### 2.2.3 Simplified Copying Style Questionnaire (SCSQ)

Coping style was measured by the 20-item Simplified Copying Style Questionnaire (Chinese version, [4]). The scale consisted of two subscales: Positive coping style (12 items) and Negative coping style (8 items). Items were measured on a 4-point Likert scale (0 = never, 1 = occasionally, 2 = sometimes, 3 = frequently). It could measure the action that people often take in daily life. The scale showed good reliability and validity [4]. In this data set, Cronbach's α of the scale was 0.794.

#### 2.2.4 Social Support Scale (SSS)

Social support was measured by the 17-item Social Support Scale (Chinese version, [5]). The scale was initially used to measure the social support of college students, but a later study [1] found that it was also applied to high school students. The scale consisted of three subscales: Subjective support (5 items), Objective support (6 items) and Support utilization (6 items). Items from this scale were rated on a 5-point Likert scale (1 = non-conformance, 2 = somewhat non-conformance, 3 = uncertainty, 4 = somewhat conformance, 5 = conformance). Higher scores usually represented higher level of the social support. The scale showed good reliability and validity [5]. In this data set, Cronbach's α of the scale was 0.920.

#### 2.2.5 Egna Minnen av Barndoms Uppforstran (EMBU)

Parenting style was measured by Egna Minnen av Barndoms Uppforstran (Chinese version, [6]). This instrument was used to evaluate parents' parenting attitudes and behaviors. EMBU consisted of two parts: Father's parenting style and Mother's parenting style, both part containing 66 items. Father's parenting style involved six dimensions: Warmth and understanding (19 items), Punishment (12 items), Overinvolved (10 items), Preference (5 items), Rejection (6 items), Overprotective (6 items) and 8 items that were not part of the six dimensions. Mother's parenting style involved five dimensions: Warmth and understanding (19 items), Overinvolved and overprotective (16 items), Rejection (8 items), Punishment (9 items), Preference (5 items) and 9 items that were not part of the five dimensions. In addition, the total scores of Warmth and understanding were seen as positive parenting style, while the total scores of other dimensions were seen as negative parenting style. Items were measured on a 4-point Likert scale (1 = never, 2 = occasionally, 3 = often, 4 = always). In this data set, Cronbach's α of the scale was 0.921.

#### 2.2.6 General Self-Efficacy Scale (GSES)

Self-efficacy was measured by the 10-item General Self-Efficacy Scale (Chinese version, [7]). GSES showed the degree of self-confidence of individuals when encountering difficulties. Items were measured on a 4-point Likert scale (1 = completely incorrect, 2 = somewhat correct, 3 = mostly correct, 4 = completely correct). Higher score indicating a higher level of self-efficacy. The reliability and validity of the scale were good. The results of Exploratory Factor Analysis showed that GSES was unidimensional. In this data set, Cronbach's α of the scale was 0.887.

### 2.3 Statistical analysis

The results of descriptive statistics (*Mean* and *SD*) and correlations among the total scores of all the variables in the questionnaires are presented in Table 5, Table 6.

## Transparency document

Transparency document associated with this article can be found in the online version at https://github.com/liutour/Teenagers-Mental-Health-in-South-China.
