# Supplementary material for: Data for teenagers' stressor, mental health, coping style, social support, parenting style and self-efficacy in South China
Source: Data Brief. 2020 Jan 25;29:105202. doi: 10.1016/j.dib.2020.105202 (PMC7011024; doi:10.1016/j.dib.2020.105202)
Supplement: Multimedia component 1 [file mmc1.zip › Supplementary material/Questionnaire (Chinese version).docx]

1. **中学生应激源量表（郑全全、陈树林，1999）**

**郑全全, 陈树林. (1999). 中学生应激源量表的初步编制. *心理发展与教育, 4,* 46-50.**

1．考试前复习紧张。

2．老师教育方式粗暴。

3．父母感情不和或家庭破裂。

4．被同学朋友误会。

5．恐怖的影视、小说、期刊等。

6．情绪不稳定。

7．考试成绩不理想。

8．被老师讽刺挖苦。

9．家里发生天灾人祸(亲人病重、死亡、受灾等)。

10．被同学朋友歧视、冷落。

11．淫秽、色情的影视、小说、期刊等。

12．意志不坚强。

13．学习任务过紧，心理负担过重。

14．老师偏心、不公正。

15．经常受父母打骂、训斥。

16．与同学朋友发生争吵、纠纷。

17．不良的社会风气(如赌博)。

18．老师教学水平低。

19．父母管教过严。

20．当众丢面子或名誉受损。

21．社会交际过多。

22．有不良习惯。

23．与老师有矛盾、关系紧张。

24．父母放任不管或过分溺爱。

25．结交异性朋友受到干涉。

26．在社会上交友不当。

27．所预期的评优落空。

28．老师作风不正。

29．父母管教方式不一致。

30．身体发生明显变化(如青春期)。

31．担心中考或高考成绩不理想。

32．被老师批评、处分。

33．父亲或母亲品行不端。

34．知心朋友很少、甚至没有。

35．睡眠不好(入睡困难、睡眠时间减少)。

36．家庭经济困难。

37．与异性朋友关系不和或决裂。

38．与家庭其它成员关系不和。

39．令人讨厌的社会习俗(如迷信活动)。

**评分标准：**

采用五级评分。其中，0＝没有发生或没有影响，1＝轻度影响，2＝中度影响，3＝重度影响，4＝极重度影响。

**量表维度：**

学习压力：1、7、13、27、31

教师压力：2、8、14、18、23、28、32

家庭环境压力：3、9 、33、36、38

父母管教方式压力：15、19、24、29

同学朋友压力：4、10、16、20、25、34、37

社会文化压力：5、11、17、21、26、39

自我身心压力：6、12、22、30、35

1. **症状自评量表（SCL-90）（王征宇，1984）**

**王征宇. (1984). 症状自评量表(SCL-90). *上海精神医学,* 2, 68-70.**

1．头痛。

2．神经过敏，心中不踏实。

3．头脑中有不必要的想法或字句盘旋。

4．头昏或昏倒。

5．对异性的兴趣减退。

6．对旁人责备求全。

7．感到别人能控制您的思想。

8．责怪别人制造麻烦。

9．忘性大。

10. 担心自己的衣饰的整齐及仪态的端正。

11．容易烦恼和激动。

12．胸痛。

13．害怕空旷的场所或街道。

14．感到自己的精力下降，活动减慢。

15．想结束自己的生命。

16．听到旁人听不到的声音。

17．发抖。

18．感到大多数入都不可信任。

19．胃口不好。

20．容易哭泣。

21．同异性相处时感害羞不自在。

22．感到受骗、中了圈套或有人想抓住您。

23．无缘无故地突然感到害怕。

24．自己不能控制地大发脾气。

25．怕单独出门。

26．经常责怪自己。

27．腰痛。

28．感到难以完成任务。

29．感到孤独。

30．感到苦闷。

31．过分担忧。

32．对事物不感兴趣。

33．感到害怕。

34．我的感情容易受到伤害。

35．旁人能知道您的私下想法。

36．感到别人不理解您、不同情您。

37．感到人们对您不友好，不喜欢您。

38．做事必须做得很慢以保证做得正确。

39．心跳得很厉害。

40．恶心或胃部不舒服。

41．感到比不上他人。

42．肌肉酸痛。

43．感到有人在监视您、谈论您。

44．难以入睡。

45．做事必须反复检查。

46．难以作出决定。

47．怕乘电车、公共汽车、地铁或火车。

48．呼吸有困难。

49，一阵阵发冷或发热。

50．因为感到害怕而避开某些东西、场合或活动。

51．脑子变空了。

52．身体发麻或刺痛。

53．喉咙有梗塞感。

54．感到没有前途、没有希望。

55．不能集中注意。

56．感到身体某一部分软弱无力。

57．感到紧张或容易紧张。

58．感到手或脚发重。

59．想到死亡的事。

60．吃得太多。

61．当别人看着您或谈论您时感到不自在。

62．有一些不属于您自己的想法。

63．有想打人或伤害他人的冲动。

64．醒得太早。

65．必须反复洗手，点数目或触摸某些东西。

66．睡得不稳不深。

67．有想摔坏或破坏东西的冲动。

68．有一些别人没有的想法或念头。

69．感到对别人神经过敏。

70．在商店或电影院等人多的地方感到不自在。

71．感到任何事情都很困难。

72．一阵阵恐惧或惊恐。

73．感到在公共场合吃东西很不舒服。

74．经常与人争论。

75．单独一人时神经很紧张。

76．别人对您的成绩没有做出恰当的评价。

77．即使和别人在一起也感到孤独。

78．感到坐立不安、心神不定。

79．感到自己没有什么价值。

80．感到熟悉的东西变成陌生或不像是真的。

81．大叫或摔东西。

82．害怕会在公共场合昏倒。

83．感到别人想占您的便宜。

84．为一些有关“性”的想法而很苦恼。

85．您认为应该为自己的过错而受到惩罚。

86．感到要赶快把事情做完。

87．感到自己的身体有严重问题。

88．从未感到和其他人很亲近。

89．感到自己有罪。

90．感到自己的脑子有毛病。

**评分标准：**

采用五级评分，0＝从无，1=轻度，2=中度，3=相当重，4＝严重。

**量表维度：**

1．躯体化(Somatization)：1、4、12、27、40、42、48、49、52、53、56、58

2．强迫症状(Obsessive-Compulsive)：3、9、10、28、38、45、46、51、55、65

3．人际关系敏感(Interpersonal sensitivity)：6、21、34、36、37、41、61、69、73

4．抑郁(Depression)：5、14、15、20、22、26、29、30、31、32、54、71、79

5．焦虑(Anxiety)：2、17、23、33、39、57、72、78、80、86

6．敌对(Hostility)；11、24、63、67、74、81

7．恐怖(Photic anxiety)：13、25、47、50、70、75、82

8．偏执(Paranoid ideation)：8、18、43、68、76、83

9．精神病性(Psychoticism)：7、16、35、62、77、84、85、87、88、90

10．其他(Additional items)：19、44、59、60、64、66、89共7个项目未归入任何因子，作为第10个因子来处理

1. **简易应对方式问卷（解亚宁，1998）**

**解亚宁. (1998). 简易应对方式量表信度和效度的初步研究. *中国临床心理学杂志, 6*(2), 114-115.**

遇到挫折打击时可能采取的态度和方法：

1. 通过工作学习或一些其他活动解脱。

2. 与人交谈，倾诉内心烦恼。

3. 尽量看到事物好的一面。

4. 改变自己的想法，重新发现生活中什么重要。

5. 不把问题看得太严重。

6. 坚持自己的立场，为自己想得到的斗争。

7. 找出几种不同的解决问题的方法。

8. 向亲戚朋友或同学寻求建议。

9. 改变原来的一些做法或自己的一些问题。

10. 借鉴他人处理类似困难情境的方法。

11. 寻求业余爱好，积极参加文体活动。

12. 尽量克制自己的失望、悔恨、悲伤或愤怒。

13. 试图休息或休假，暂时把问题（烦恼）抛开。

14. 通过吸烟、喝酒、服药和吃东西来解除烦恼。

15. 认为时间会改变现状，唯一要做的便是等待。

16. 试图忘记整个事情。

17. 依靠别人解决问题。

18. 接受现实，因为没有其它办法。

19. 幻想可能会发生某种奇迹改变现状。

20. 自己安慰自己。

**评分标准：**

采用四级评分，其中，0=不采取，1=偶尔采取，2=有时采取，3=经常采取。

**量表维度：**

积极应对：1-12

消极应对：13-20

1. **社会支持量表（叶悦妹、戴晓阳，2008）**

**叶悦妹, 戴晓阳. (2008). 大学生社会支持评定量表的编制. *中国临床心理学杂志, 16*(5), 456-458.**

1. 大多数同学都很关心我。

2. 面对两难的选择时，我会主动向他人寻求帮助。

3. 当有烦恼时，我会主动向家人、亲友倾诉。

4. 我经常能得到同学、朋友的照顾和支持。

5. 当遇到困难时，我经常会向家人、亲人寻求帮助。

6. 我周围有许多关系密切、可以给予我支持和帮助的人。

7. 在我遇到困难时，同学、朋友会出现在我身旁。

8. 在困难的时候，我可以依赖家人或亲友。

9. 我经常从同学、朋友那里获得情感上的帮助和支持。

10. 我经常能得到家人、亲友的照顾和支持。

11. 需要时，我可以从家人、亲友那里得到经济支持。

12. 当遇到麻烦时，我通常会主动寻求别人的帮助。

13. 当我生病时，总能得到家人、亲友的照顾。

14. 当有烦恼时，我会主动向同学、朋友倾诉。

15. 在我遇到问题时，家人、亲友会出现在我身旁。

16. 我经常从家人、亲友那里获得情感上的帮助和支持。

17. 当遇到困难时，我经常会向同学、朋友寻求帮助。

**评分标准：**

采用五级评分，其中，1=不符合，2=有点不符合，3=不确定，4=有点符合，5=符合。

**量表维度：**

主观支持：1、4、6、7、9

客观支持：8、10、11、13、15、16

支持利用度：2、3、5、12、14、17

1. **父母教养方式评价量表（岳冬梅、李鸣杲、金魁和、丁宝坤，1993）**

**岳冬梅, 李鸣杲, 金魁和, 丁宝坤. (1993). 父母教养方式: EMBU的初步修订及其在神经症患者的应用. *中国心理卫生杂志, 7*(3), 97-101.**

1. 我觉得我父母干涉我所做的任何一件事。

2. 我能通过父母的言谈、表情感受他(她)很喜欢我。

3. 与我的兄弟姐妹相比，父母更宠爱我。

4. 我能感到父母对我的喜爱。

5. 即使是很小的过失，父亲也惩罚我。

6. 父母总试图潜移默化地影响我，使我成为出类拔萃的人。

7. 我觉得父母允许我在某些方面有独到之处。

8. 父母能让我得到其他兄弟姐妹得不到的东西。

9. 父母对我的惩罚是公平的。

10. 我觉得父亲对我很严厉。

11. 父母总是左右我该穿什么衣服或该打扮成什么样子。

12. 父母不允许我做一些其他孩子可以做的事情，因为他们害怕我会出事。

13. 在我小时候，父母曾经当着别人面打我或训斥我。

14. 父母总是很关心我晚上干什么。

15. 当遇到不顺心的事时，我能感到父母在尽量鼓励我，使我得到一些安慰。

16. 父母总是过分担心我的健康。

17. 父母对我的惩罚往往超过我应受的程度。

18. 如果我在家里不听吩咐，父亲就会恼火。

19. 如果我做错了什么事，母亲总是以一种伤心的样子使我有一种犯罪感或内疚感。

20. 我觉得父亲难以接近。

21. 父亲曾在别人面前唠叨一些我说过的话或做过的事，这使我感到很难堪。

22. 我觉得父母更喜欢我，而不是我的兄弟姐妹。

23. 在满足我需要的东西，父母是很小气的。

24. 母亲常常很在乎我取得的分数。

25. 如果我面临一项困难的任务，我能感到来自父母的支持。

26. 我在家里往往被当作"替罪羊"或"害群之马"。

27. 父母总是挑剔我所喜欢的朋友。

28. 父母总以为他们的不快是由我引起的。

29. 父母总试图鼓励我，使我成为佼佼者。

30. 父母总向我表示他们是爱我的。

31. 父母对我很信任，且允许我独自完成某些事。

32. 我觉得父母很尊重我的观点。

33. 我觉得父母很愿意跟我在一起。

34. 我觉得父母对我很小气、很吝啬。

35. 父母总是向我说类似这样的话“如果你这样做我会很伤心”。

36. 父母要求我回到家里必须得向他们说明我在做的事情。

37. 我觉得父母在尽量使我的青春更有意义和丰富多彩（如给我买很多的书，安排我去夏令营或参加俱乐部）。

38. 母亲经常向我表述类似这样的话“这就是我们为你整日操劳而得到的报答吗？”

39. 父母常以不能娇惯我为借口不满足我的要求。

40. 如果不按父亲所期望的去做，就会使我在良心上感到不安。

41. 我觉得母亲对我的学习成绩、体育活动或类似的事情有较高的要求。

42. 当我感到伤心的时候可以从父母那儿得到安慰。

43. 父母曾无缘无故地惩罚我。

44. 父母允许我做一些我的朋友们做的事情。

45. 父母经常对我说他们不喜欢我在家的表现。

46. 每当我吃饭时，父母就劝我或强迫我再多吃一些。

47. 母亲经常当着别人的面批评我既懒惰，又无用。

48. 父母常常关注我交什么样的朋友。

49. 如果发生什么事情，我常常是兄弟姐妹中唯一受责备的一个。

50. 父母能让我顺其自然地发展。

51. 父母经常对我粗俗无礼。

52. 有时甚至为一点儿鸡毛蒜皮的小事，父母也会严厉地惩罚我。

53. 父母曾无缘无故地打我。

54. 母亲通常会参与我的业余爱好活动。

55. 我经常挨父母的打。

56. 父母常允许我到我喜欢去的地方，而他们又不会过分担心。

57. 父母对我该做什么、不该做什么都有严格的限制而绝不让步。

58. 父母常以一种使我很难堪的方式对待我。

59. 我觉得父母对我可能出事的担心是夸大的、过分的。

60. 我觉得与父母之间存在一种温暖、体贴和亲热的感觉。

61. 父母能容忍我与他们有不同的见解。

62. 父母常常在我不知道原因的情况下对我大发脾气。

63. 当我做的事情取得成功时，我觉得母亲为我很自豪。

64. 与我的兄弟姐妹相比，父母常常偏爱我。

65. 有时即使错误在我，父母也把责任归咎于兄弟姐妹。

66. 父亲经常拥抱我。

**评分标准：**

采用四级评分，1=从不，2=有时，3=经常，4=总是

**父亲教养方式**（共58条目）

需要反向计分的项目有：20、50、56（在数据中已经进行了反向计分）

情感温暖、理解：2、4、6、7、9、15、20、25、29、30、31、32、33、37、42、44、60、61、66

惩罚、严厉：5、13、17、18、43、49、51、52、53、55、58、62

过分干涉：1、10、11、14、27、36、48、50、56、57

偏爱被试：3、8、22、64、65

拒绝、否认：21、23、28、34、35、45

过度保护：12、16、39、40、46、59

父亲教养方式中不包含项目：19、24、26、38、41、47、54、63

**母亲教养方式**（共57 条目）

需要反向计分的项目有：50、56（在数据中已经进行了反向计分）

情感温暖、理解：2、4、6、7、9、15、25、29、30、31、32、33、37、42、44、54、60、61、63

过分干涉、过度保护：1、11、12、14、16、19、24、27、35、36、41、48、50、56、57、59

拒绝、否认：23、26、28、34、38、39、45、47

惩罚、严厉：13、17、43、51、52、53、55、58、62

偏爱被试：3、8、22、64、65

母亲教养方式中不包含项目：5、10、18、20、21、40、46、49、66

1. **自我效能感量表（王才康、胡中锋、刘勇，2001）**

**王才康,胡中锋,刘勇. (2001). 一般自我效能感量表的信度和效度研究. *应用心理学, 7*(1), 37-40.**

1. 如果我尽力去做的话，我总是能够解决问题的。

3. 对我来说，坚持理想和达成目标是轻而易举的。

2. 即使别人反对我，我仍有办法取得我所要的。

4. 我自信能有效地应付任何突如其来的事情。

5. 以我的才智，我定能应付意料之外的情况。

6. 如果我付出必要的努力，我一定能解决大多数的难题。

7. 我能冷静地面对困难，因为我可信赖自己处理问题的能力。

8. 面对一个难题时，我通常能找到几个解决方法。

9. 有麻烦的时候，我通常能想到一些应付的方法。

10. 无论什么事在我身上发生，我都能够应付自如。

**评分标准：**

采用四级评分，其中，1=完全不正确，2=有点正确，3=多数正确，4=完全正确。

**量表维度（单维）：**

自我效能感：1-10
