# Supplementary material for: Data for teenagers' stressor, mental health, coping style, social support, parenting style and self-efficacy in South China
Source: Data Brief. 2020 Jan 25;29:105202. doi: 10.1016/j.dib.2020.105202 (PMC7011024; doi:10.1016/j.dib.2020.105202)
Supplement: Multimedia component 1 [file mmc1.zip › Supplementary material/Questionnaire (English version).docx]

**① Stressors Scale for Middle School Students (SSMSS)** (Zheng & Chen, 1999)

Zheng, Q. Q., & Chen, S.L. (1999). Preliminary revision of Stressor Scale for High School Students, *Psychological Development and Education. 15*(4), 45-49.

1. Feel nervous before the exam.

2. The way of teacher’s education is rough.

3. Parents' discord or family breakdown.

4. Misunderstood by classmates and friends.

5. Horror films, novels, periodicals, etc.

6. Emotional instability.

7. The examination result is not ideal.

8. Be satirized by the teacher.

9. Natural and man-made disasters occur at home (serious illness, death, disaster, etc).

10. Discriminated and neglected by classmates and friends.

11. Pornographic movies, novels, periodicals, etc.

12. The will is not determined.

13. The study task is too tight and the psychological burden is too heavy.

14. Teachers are biased and unfair.

15. Be beaten and scolded by parents.

16. Quarrel and dispute with classmates and friends.

17. Bad social atmosphere (such as gambling).

18. Teachers' teaching level is low.

19. Strict parental discipline.

20. Lose face or reputation in public.

21. Too much social communication.

22. Have bad habits.

23. There are contradictions and tensions with teachers.

24. Parents indulge or overindulge.

25. Interference in making friends of the opposite sex.

26. Making friends improperly in society.

27. The expected appraisal was lost.

28. There is a problem with the teacher's behavior style.

29. The ways of parents' discipline are inconsistent.

30. Physical changes (such as adolescence).

31. Worry about poor scores in the high school entrance examination or the college entrance examination.

32. Be criticized and punished by the teacher.

33. A father or mother has bad conduct.

34. Few or no close friends.

35. Poor sleep (difficulty in falling asleep, reduced sleep time).

36. Family financial difficulties.

37. Disagreement or break up with friends.

38. Disharmony with other members of the family.

39. Objectionable social practices (such as superstitious activities).

**Scoring criteria:**

Items were measured on a 5-point Likert scale (0 = no effect, 1 = slight effect, 2 = moderate effect, 3 = severe effect, 4 = extremely severe effect).

**Scale dimensions:**

Learning stress: 1, 7, 13, 27, 31

Teacher stress: 2, 8, 14, 18, 23, 28, 32

Family environment stress: 3, 9, 33, 36, 38

Parenting style stress: 15, 19, 24, 29

Classmates and friends stress: 4, 10, 16, 20, 25, 34, 37

Social and culture stress: 5, 11, 17, 21, 26, 39

Physical and psychological stress: 6, 12, 22, 30, 35

**② Symptom Check-List 90 (SCL90)** (Wang, 1984)

Wang, Z.Y. (1984). Symptom Checklist (SCl-90), *Shanghai Archives of Psychiatry.* 2, 68-70.

1. Headache.

2. Nervous and unstable.

3. There are unnecessary thoughts or words in your mind.

4. Dizziness or fainting.

5. Decreased interest in the opposite sex.

6. Embarrass others.

7. Feel that others can control your mind.

8. Blame others for making trouble.

9. Forgetfulness.

10. Worry about the tidiness of their clothes and their manners.

11. Easily upset and excited.

12. Chest pain.

13. Fear of the empty places or streets.

14. Feel your energy and activity decrease.

15. Want to end your life.

16. Hear a voice that no one else can hear.

17. Shivering.

18. Feel that others are untrustworthy.

19. Bad appetite.

20. Easy to cry.

21. Feel shy and uncomfortable with the opposite sex.

22. Feel cheated, trapped or someone wants to catch you.

23. Suddenly feel afraid for no reason.

24. Lose your temper uncontrollably.

25. Afraid to go out alone.

26. Always blame yourself.

27. Back ache.

28. Find it difficult to complete your work.

29. Feel lonely.

30. Feel depressed.

31. Worry too much.

32. Not interested in things.

33. Afraid.

34. My feelings are vulnerable.

35. Others can know your private thoughts.

36. Feel that others don't understand you and don't love you.

37. Feel that people are not friendly to you and don't like you.

38. Do things slowly to make sure they are done right.

39. The heart beat very hard.

40. Nausea or stomach discomfort.

41. Feel inferior to others.

42. Muscle ache.

43. Feel someone watching you and talking about you.

44. Difficult to sleep.

45. Do things repeatedly.

46. It is difficult to make a decision.

47. Afraid to take tram, bus, subway or train.

48. Difficulty breathing.

49. Chills or fever.

50. Avoid something, an occasion, or an activity because you are afraid.

51. The brain is empty.

52. Body tingling or tingling.

53. The throat has a feeling of infarction.

54. Feeling hopeless.

55. Can't concentrate.

56. Feel a part of your body weak.

57. Feel nervous or easily nervous.

58. Feel your hands or feet are heavy.

59. Think of death.

60. Eat too much.

61. Feel uncomfortable when others look at you or talk about you.

62. There are some ideas that do not belong to you.

63. Have the impulse to hit or hurt others.

64. Wake up too early.

65. Wash your hands repeatedly or touch something.

66. Can't sleep very well.

67. Have the impulse to break or destroy something.

68. There are some ideas or thoughts that others don't have.

69. Feel nervous about others.

70. Feel uneasy in places like shops or cinemas.

71. Feel that everything is difficult.

72. A wave of fear or panic.

73. Feel uncomfortable when eating in public.

74. Often argue with others.

75. Feel nervous when alone.

76. Others don't give proper evaluation of your achievements.

77. Feel lonely even with others.

78. Feel restless and uneasy.

79. Feeling worthless.

80. Things that are familiar before become strange or not true.

81. Shout or drop something.

82. Fear of fainting in public.

83. Feel that others want to take advantage of you.

84. Distressed by some ideas about sex.

85. You think you should be punished for your fault.

86. Feel like you need to get things done quickly.

87. Feel that you have serious physical problems.

88. Never feel close to others.

89. Feel guilty.

90. Feel your brain is out of order.

**Scoring criteria:**

Items from this scale were rated on a 5-point Likert scale (0 = never, 1 = slight, 2 = moderate, 3 = heavy, 4 = serious).

**Scale dimensions:**

Somatization: 1, 4, 12, 27, 40, 42, 48, 49, 52, 53, 56, 58

Obsessive-compulsive: 3, 9, 10, 28, 38, 45, 46, 51, 55, 65

Interpersonal sensitivity: 6, 21, 34, 36, 37, 41, 61, 69, 73

Depression: 5, 14, 15, 20, 22, 26, 29, 30, 31, 32, 54, 71, 79

Anxiety: 2, 17, 23, 33, 39, 57, 72, 78, 80, 86

Hostility; 11, 24, 63, 67, 74, 81

Photic anxiety: 13, 25, 47, 50, 70, 75, 82

Paranoid ideation: 8, 18, 43, 68, 76, 83

Psychoticism: 7, 16, 35, 62, 77, 84, 85, 87, 88, 90

Additional items: 7 items (19, 44, 59, 60, 64, 66, 89) are not included in any factors, and are treated as the 10th factor.

**③ Simplified Copying Style Questionnaire (SCSQ)** (Xie, 1998)

Xie, Y. N. (1998). Preliminary study on reliability and validity of Simplified Coping Style Questionnaire, *Chinese Journal of Clinical Psychology. 6*(2), 114-115.

Possible attitudes and methods you will take when you encounter some troubles:

1. Free from work, study or other activities.

2. Talk to people and talk about your troubles.

3. Try to see the good side of things.

4. Change your mind and rediscover what's important in life.

5. Don't take the problem too seriously.

6. Stick to your position and fight for what you want.

7. Find out several different ways to solve the problem.

8. Seek advice from relatives, friends or classmates.

9. Change some of the original practices or some of your own problems.

10. Learn from others to deal with similar difficult situations.

11. Seek hobbies and actively participate in cultural and sports activities.

12. Try to control your disappointment, regret, sadness or anger.

13. Try to take a rest or vacation and put aside the problems (troubles) temporarily.

14. Relieve worry by smoking, drinking, taking medicine and eating.

15. Think that time will change the situation, just need to wait.

16. Try to forget the whole thing.

17. Rely on others to solve problems.

18. Accept the reality because there is no other way.

19. Fantasize that some things will happen to change the situation.

20. Comfort yourself.

**Scoring criteria:**

Items were measured on a 4-point Likert scale (0 = never, 1 = occasionally, 2 = sometimes, 3 = frequently).

**Scale dimensions:**

Positive coping style: 1-12

Negative coping style: 13-20

**④ Social Support Scale (SSS)** (Ye & Dai, 2008)

Ye, Y.M., & Dai, X.Y. (2008). Development of Social Support Scale for College Students, *Chinese Journal of Clinical Psychology. 16*(5), 456-458.

1. Most of my classmates are very concerned about me.

2. In the face of a dilemma, I will actively seek help from others.

3. When there are troubles, I will talk to my family and friends.

4. I often get the care and support of my classmates and friends.

5. When I encounter difficulties, I often ask my family and relatives for help.

6. I am surrounded by many people who are closely related and can give me support and help.

7. When I encounter difficulties, my classmates and friends will appear.

8. In difficult times, I can rely on my family or friends.

9. I often get emotional help and support from my classmates and friends.

10. I often get the care and support of my family and friends.

11. I can get financial support from my family and friends when I need it.

12. When in trouble, I usually ask for help.

13. When I am ill, I always get the care of my family and friends.

14. When there are troubles, I will talk to my classmates and friends.

15. When I have problems, my family and friends will appear.

16. I often get emotional help and support from my family and friends.

17. When I encounter difficulties, I often ask my classmates and friends for help.

**Scoring criteria:**

Items from this scale were rated on a 5-point Likert scale (1 = non-conformance, 2 = somewhat non-conformance, 3 = uncertainty, 4 = somewhat conformance, 5 = conformance).

**Scale dimensions:**

Subjective support: 1, 4, 6, 7, 9

Objective support: 8, 10, 11, 13, 15, 16

Support utilization: 2, 3, 5, 12, 14, 17

**⑤ Egna Minnen av Barndoms Uppforstran (EMBU)** (Yue, Li, Jin, & Ding, 1993)

Yue, D.M., Li, M.G., Jin, K.H., & Ding, B.K. (1993). Parenting style: Preliminary revision of EMBU and its application in neurosis patients, *Chinese Mental Health Journal. 7*(3), 97-101.

1. I think my parents interfere in everything I do.

2. I can feel that my parents like me very much through their words and expressions.

3. Compared with my brothers and sisters, my parents prefer me.

4. I can feel my parents love me.

5. My father punished me even for minor mistakes.

6. My parents always try to influence me imperceptibly and make me outstanding.

7. I think my parents allow me to be unique in some aspects.

8. My parents can let me get what other brothers and sisters can't get.

9. My parents' punishment is fair.

10. I think my father is very strict with me.

11. My parents always decide what I should wear or what I should look like.

12. My parents don't allow me to do something that other children can do, because they are afraid that something will happen to me.

13. When I was a child, my parents beat me or scolded me in front of others.

14. My parents are always concerned about what I do at night.

15. When something goes wrong, I can feel my parents encourage me as much as possible, so that I can get some comfort.

16. My parents always worry too much about my health.

17. My parents often punish me more than I should.

18. If I am not obedient, my father will be angry.

19. If I do something wrong, my mother always makes me feel guilty or guilty.

20. I find it hard to get close with my father.

21. I was embarrassed by my father's nagging in front of others about what I said or did.

22. I think my parents prefer me to my brothers and sisters.

23. My parents are stingy in satisfying my needs.

24. My mother often cares about my scores.

25. If I face a difficult task, I can feel the support from my parents.

26. I am often regarded as a "scapegoat" or "black sheep" at home.

27. My parents are always critical of my favorite friends.

28. Parents always think that their unhappiness is caused by me.

29. My parents always try to encourage me to be the best.

30. My parents always show me that they love me.

31. My parents trust me very much and allow me to accomplish something by myself.

32. I think my parents respect my point of view.

33. I think my parents would like to be with me.

34. I think my parents are stingy to me.

35. My parents always say something like "if you do this, I will be very sad".

36. My parents ask me to tell them what I am doing when I come home.

37. I think my parents are trying to make my youth more meaningful and colorful (such as buying me a lot of books, arranging me to go to summer camp or join a club).

38. My mother often said to me something like, "Is this the reward we get for work hard all day for you?"

39. My parents often refuse to satisfy my needs on the pretext that they can't pamper me.

40. If I don't do what my father expected, I will feel uneasy.

41. I think my mother has strict standards for my academic performance, sports activities or similar things.

42. I can get comfort from my parents when I feel sad.

43. My parents punished me for nothing.

44. My parents allow me to do something my friends do.

45. My parents often tell me that they don't like my performance at home.

46. When I eat, my parents want me to eat more.

47. My mother often criticizes me for being lazy and useless in front of others.

48. My parents often pay attention to what kind of friends I make.

49. If something happens, I am the only one to be blamed rather than my brothers and sisters.

50. My parents can let me develop naturally.

51. My parents are often rude to me.

52. Sometimes my parents will punish me severely even for trifles.

53. My parents hit me for no reason.

54. My mother usually takes part in my hobbies.

55. I am often beaten by my parents.

56. My parents often allow me to go where I like and they don't worry too much.

57. My parents have strict restrictions on what I should do and what I should not do.

58. My parents often treat me in a way that embarrasses me.

59. I think my parents' worry about my possible accident is exaggerated and excessive.

60. I feel a warm, considerate and intimate feeling with my parents.

61. My parents can tolerate that I have different opinions from them.

62. My parents often get angry with me but I don't know why.

63. When I succeed in what I do, I feel that my mother is very proud of me.

64. Compared with my brothers and sisters, my parents prefer me.

65. Sometimes my parents blame my brothers and sisters for my mistakes.

66. My father often hugs me.

**Scoring criteria:**

Items were measured on a 4-point Likert scale (1 = never, 2 = occasionally, 3 = often, 4 = always).

**Father's parenting style (58 items in total)**

Items requiring reverse scoring are: 20, 50, 56 (reverse scoring has been performed in the data)

Warmth and understanding: 2, 4, 6, 7, 9, 15, 20, 25, 29, 30, 31, 32, 33, 37, 42, 44, 60, 61, 66

Punishment: 5, 13, 17, 18, 43, 49, 51, 52, 53, 55, 58, 62

Overinvolved: 1, 10, 11, 14, 27, 36, 48, 50, 56, 57

Preference: 3, 8, 22, 64, 65

Rejection: 21, 23, 28, 34, 35, 45

Overprotective: 12, 16, 39, 40, 46, 59

8 items that were not part of the six dimensions: 19, 24, 26, 38, 41, 47, 54, 63

**Mother's parenting style (57 items in total)**

Items requiring reverse scoring are: 50, 56 (reverse scoring has been performed in the data)

Warmth and understanding: 2, 4, 6, 7, 9, 15, 25, 29, 30, 31, 32, 33, 37, 42, 44, 54, 60, 61, 63

Overinvolved and overprotective: 1, 11, 12, 14, 16, 19, 24, 27, 35, 36, 41, 48, 50, 56, 57, 59

Rejection: 23, 26, 28, 34, 38, 39, 45, 47

Punishment: 13, 17, 43, 51, 52, 53, 55, 58, 62

Preference: 3, 8, 22, 64, 65

9 items that were not part of the five dimensions: 5, 10, 18, 20, 21, 40, 46, 49, 66

**⑥ General Self-Efficacy Scale (GSES)** (Wang, Hu, & Liu,2001)

Wang, C.K., Hu, Z.F., & Liu, Y. (2001). Study on reliability and validity of General Self-Efficacy Scale, *Chinese Journal of Applied Psychology. 7*(1), 37-40.

1. If I try my best, I can always solve the problem.

3. It's easy for me to stick to my ideals and achieve my goals.

2. Even if other people are against me, I still have a way to get what I want.

4. I am confident that I can deal with anything unexpected effectively.

5. With my intelligence, I will be able to cope with unexpected situations.

6. If I make the necessary efforts, I will be able to solve most of the problems.

7. I can face difficulties calmly because I can trust my ability to deal with problems.

8. When facing a difficult problem, I can usually find several solutions.

9. When I have trouble, I can usually think of some ways to deal with it.

10. No matter what happens to me, I can handle it freely.

**Scoring criteria:**

Items were measured on a 4-point Likert scale (1 = completely incorrect, 2 = somewhat correct, 3 = mostly correct, 4 = completely correct).

**Scale dimension (unidimensional):**

Self-efficacy: 1-10
